# Consequences of using poly-ether-ether-ketone versus traditional implant on tibial cement penetration and short-term clinical outcomes during total knee arthroplasty: a randomized controlled trial

**DOI:** 10.1186/s13018-023-04064-1

**Published:** 2023-08-09

**Authors:** Guanghui Zhao, Shuxin Yao, Xiangxiang Sun, Jianbing Ma, Jianpeng Wang

**Affiliations:** Department of Joint Surgery, Honghui Hospital, Xi’An Jiaotong University, No.555 East Youyi Road, Xi’an, Shanxi, China

**Keywords:** TKA, PEEK, Bone cement penetration

## Abstract

**Background:**

The use of poly-ether-ether-ketone (PEEK) prosthesis during total knee arthroplasty (TKA) is a relatively new concept. Several studies have suggested that the thickness of cement penetration during TKA may affect the stability of the implants. The present study aimed to compare the cement penetration and clinical performance between PEEK and traditional cobalt chromium molybdenum (CoCrMo) prosthesis during TKA.

**Methods:**

This study was a randomized controlled trial with level I of evidence. A total of 48 patients were randomly assigned to either the PEEK group (*n* = 24) or the CoCrMo group (*n* = 24). Mean bone cement penetration under the tibial baseplate was assessed radiographically in four zones in the anteroposterior view and two zones in the lateral view, in accordance with the Knee Society Scoring System. Furthermore, parameters such as the Knee Society Score (KSS), visual analogue scale (VAS) scores, complications and survivorship at 1 year postoperatively were also compared.

**Results:**

According to the results of this study, the mean bone cement penetration exhibited no significant difference between PEEK and CoCrMo groups (2.49 ± 0.61 mm vs. 2.53 ± 0.68 mm, *p* = 0.85). Additionally, there were no remarkable differences in the KSS clinical score, functional score, and VAS score between the two groups. Moreover, complications and survivorship were also statistically compared between the groups and presented no significant differences.

**Conclusions:**

Based on the current findings, it can be concluded that PEEK implant present similar bone cement penetration, short-term clinical outcomes, and survivorship with traditional CoCrMo implant in TKA without added complications.

*Trial registration* Chinese Clinical Trial Registry (ChiCTR2100047563).

## Background

Total knee arthroplasty (TKA) is commonly utilized to treat osteoarthritis, although aseptic loosening of the tibial component remains a primary concern [1, 2]. The durability of the TKA hinges on the strength of the cement-bone bond, particularly the ability of the cement to infiltrate the cancellous bone beneath the tibial component homogeneously [3–6]. Previous investigations have revealed that the effectiveness of cement penetration is dependent on multiple factors, including the type of cement and cementation technique employed, as well as the tourniquet use or not [7–9].

In order to increase the longevity of joint prostheses, researchers have explored various materials including cobalt chromium molybdenum, titanium, ceramic, and more recently, poly-ether-ether-ketone (PEEK) [10, 11]. PEEK offers several advantages over traditional materials, such as an elastic modulus more similar to bone, improved biocompatibility, and the ability to promote osteogenesis around the implant [12–14]. PEEK has already been used in orthopedic surgery for intervertebral lumbar cages, screws, and cranial patches [15]. However, there is little research on PEEK as an artificial knee material, with only a recent clinical trial using PEEK femoral components and all-polyethylene tibial components reported [16, 17]. There are currently no clinical studies on the use of a totally modular PEEK knee joint prosthesis.

The cement penetration depth is an important indicator of the quality and longevity of TKA. According to previous reports, cement penetration in well-performing TKA ranges from 2 to 4 mm [18–20]. With the increasing use of PEEK as a new material for knee prostheses, it is unclear whether there are differences in cement penetration depth between PEEK and conventional cobalt chromium molybdenum (CoCrMo) materials with different elastic modulus. It has been suggested that the mode of force transmission through the prosthesis is the critical factor that affects cement penetration depth. Specifically, in the case of a CoCrMo knee prosthesis, the force transmitted to the cement can cause the cement to penetrate to the ideal 2–4 mm, whereas it remains unknown whether the same cement penetration can be achieved with a PEEK material prosthesis. Therefore, a comprehensive evaluation of the mechanical properties and clinical outcomes of PEEK prostheses is needed to better understand its effects on cement penetration and short-term performance in TKA.

This study aimed to compare the effects of PEEK and CoCrMo knee prosthesis on cement penetration in the tibial bone and evaluate the short-term clinical outcomes and safety of PEEK TKA.

## Methods

From June 2021 to December 2022, a prospective randomized clinical trial was conducted at Honghui Hospital affiliated to Xi'An Jiaotong University to investigate the safety and efficacy of PEEK knee prostheses for TKA. The study was approved by the local ethics committee (NO. 2021-008-001) and registered at Chinese Clinical Trial Registry (ChiCTR2100047563) in accordance with the Helsinki Declaration. All patients provided written consent prior to enrollment.

Patients in the age range of 50–80 years who met the inclusion criteria and were planned for elective primary TKA were enrolled in this study. The inclusion criteria were clinical diagnosis of osteoarthritis, rheumatoid arthritis, traumatic arthritis, or ischemic osteonecrosis. The exclusion criteria were neuromuscular insufficiency, comprehension disorders, alcoholism, drug abuse, substance abuse, BMI > 35 kg/m^2^, known allergy to implant materials, infection of the knee or other sites, severe osteoporosis, bone disease or bone tumors, deep-vein thrombosis (DVT) of the lower extremities, or other systemic diseases that cannot tolerate the procedure.

In this study, patients were block randomized using sealed envelopes to ensure unbiased allocation into two groups: PEEK knee implants (PEEK group) and CoCrMo knee implants (CoCrMo group). The envelopes were opened in the presence of the surgeon, just before surgery in the operating theater. The patients were kept unaware of the group to which they had been allocated, in order to avoid any potential biases.

### Surgical technique

All procedures were strictly standardized in accordance with preoperative tranexamic acid (TXA), general anesthesia, postoperative pain management, and rehabilitation regimen. Prior to surgery and immediately before skin incision, TXA (1 g) and cefuroxime (1.5 g) were administered intravenously. Following joint capsule suturing, an intra-articular injection of TXA (2 g) was given. Moreover, TXA (0.5 g) was given 3 and 6 h postoperatively, with two doses of cefuroxime (1.5 g) administered within 24 h postoperatively. Thrombosis prophylaxis was achieved using low-molecular-weight heparin calcium (40AXaIU/kg per day) during hospitalization. Both groups underwent appropriate thigh tourniquet application, limb exsanguination through elevation for 2 min, and cuff inflation to 250 mmHg just prior to skin incision.

**Fig. 1:**
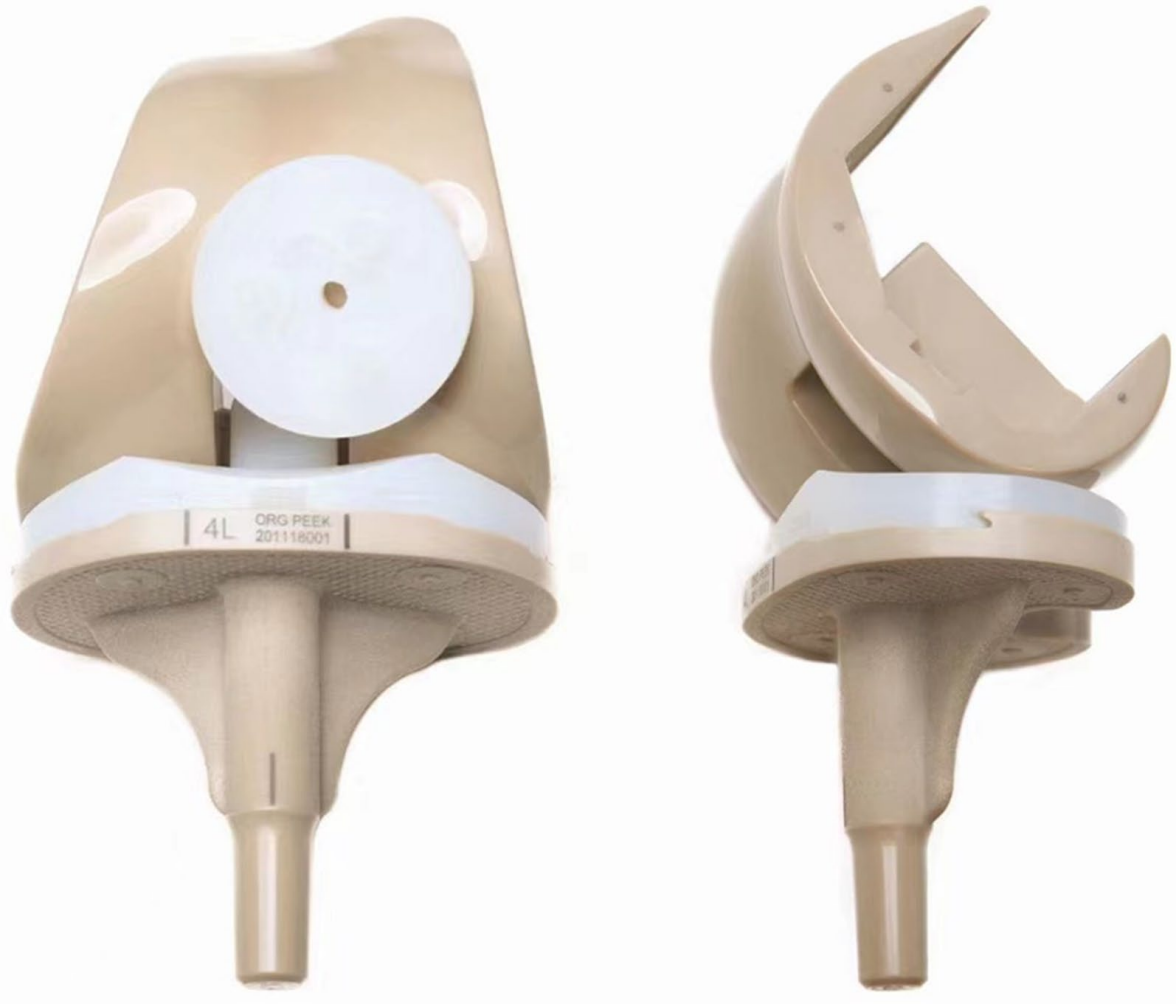
The complete peek knee system, including femoral component, tibial component, polyethylene liner, and patellar component

Knee implants were utilized in the experiment where the PEEK group consisted of the post-stabilized fixed-bearing knee from Suzhou SinoMed Biomaterials Co., Ltd. in Jiangsu, China (Fig. 1). The CoCrMo group consisted of the Genesis II post-stabilized fixed bearing knee from Smith & Nephew Orthopedics in Memphis, TN, USA. Low-viscosity bone cement, gentamicin bone cement from Heraeus in Wertheim, Germany, was used in both groups. The patella was not resurfaced in any of the cases, and the surgical procedures were performed by a group of surgeons. A midline skin incision and medial parapatellar arthrotomy were applied, along with an intramedullary guide system for the femur and external guides for the tibia.

The cementation was performed in two stages, with the tibia being implanted first, followed by fixation of the femoral component using another batch of cement. This approach allowed for adequate time to achieve meticulous cementation with proper pressurization. Following cementation, further pulse lavage debridement was carried out to eliminate cement debris from the wound. Upon closure of the joint capsule, the cuff was deflated and bipolar coagulation was utilized to halt bleeding in the subcutaneous vessels. Postoperative rehabilitation and pain management were standardized for both groups and adhered to a uniform protocol that included full weight-bearing.

**Fig. 2:**
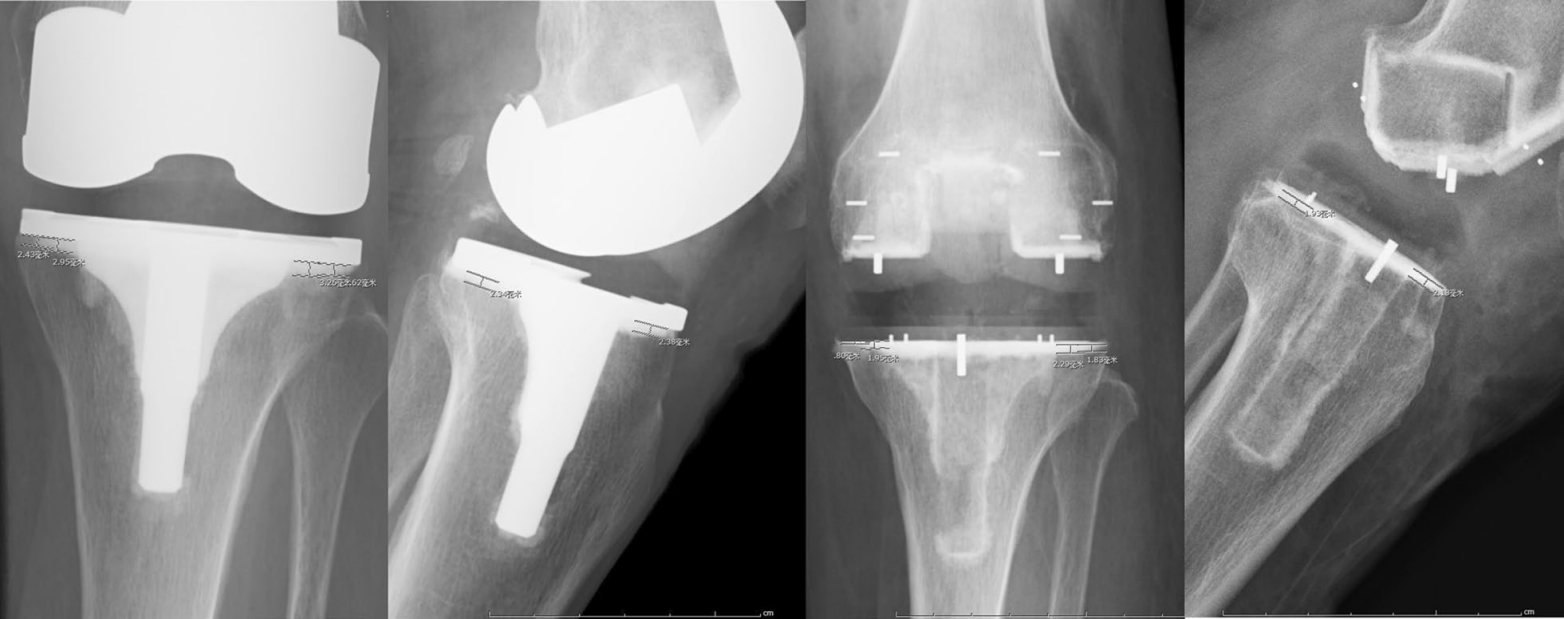
Anteroposterior and lateral radiographs with six zone cement mantle measurements

The depth of the cement penetration was assessed using the method described by Pfitzner et al. [2], based on anteroposterior and lateral knee radiographs obtained on the second day postoperatively. These radiographs were taken in the office with the patient standing upright and the knee in full extension by trained radiology technicians, following a standard format. The Knee Society Scoring System was used to divide the tibial plateau into six zones, with four zones based on the anteroposterior view and two zones on the lateral view (Fig. 2) [21]. Since the femoral component may affect the measurement of cement and the stem design varies significantly between different implants, measurements of the cement were only taken at the baseplate. The PACS measurement tool was used to measure the depth of the cement penetration.

In this study, the postoperative knee score was assessed by the use of Knee Society Score (KSS), which includes both knee clinical score and functional score, and survivorship at 1 year after surgery. Pain intensity was measured using the visual analogue scale (VAS) on a scale ranging from 0 to 10, both pre-operatively and at 1 year postoperatively during full weight-bearing mobilization of the patients. Any incidences of postoperative delayed wound healing, periprosthetic joint infection (PJI), pulmonary embolism (PE), DVT, periprosthetic joint fracture (PJF), and radiolucent lines (RLLs) was recorded and analyzed.

### Statistical analysis

Sample size was based on earlier studies, in which the priori power analysis was undertaken for an unpaired Student’s t-test, α at 0.05 and β at 0.2, i.e., Power (1 − β) or 0.8. A difference of 0.75 mm in the mean cement penetration between each group was determined to be clinically relevant. With a two-sided test, 58 patients were required. With a one-sided test, a loss of 12 patients could be tolerated [22, 23]. Statistical analysis was performed using SPSS version 25. Normally distributed data were summarized using mean and standard deviation (SD), while non-parametric data were summarized using median and range. Student's t-test was employed for parametric data, while Chi-squared test was used for non-parametric data. A p-value of less than 0.05 was considered statistically significant.

## Results

**Fig. 3:**
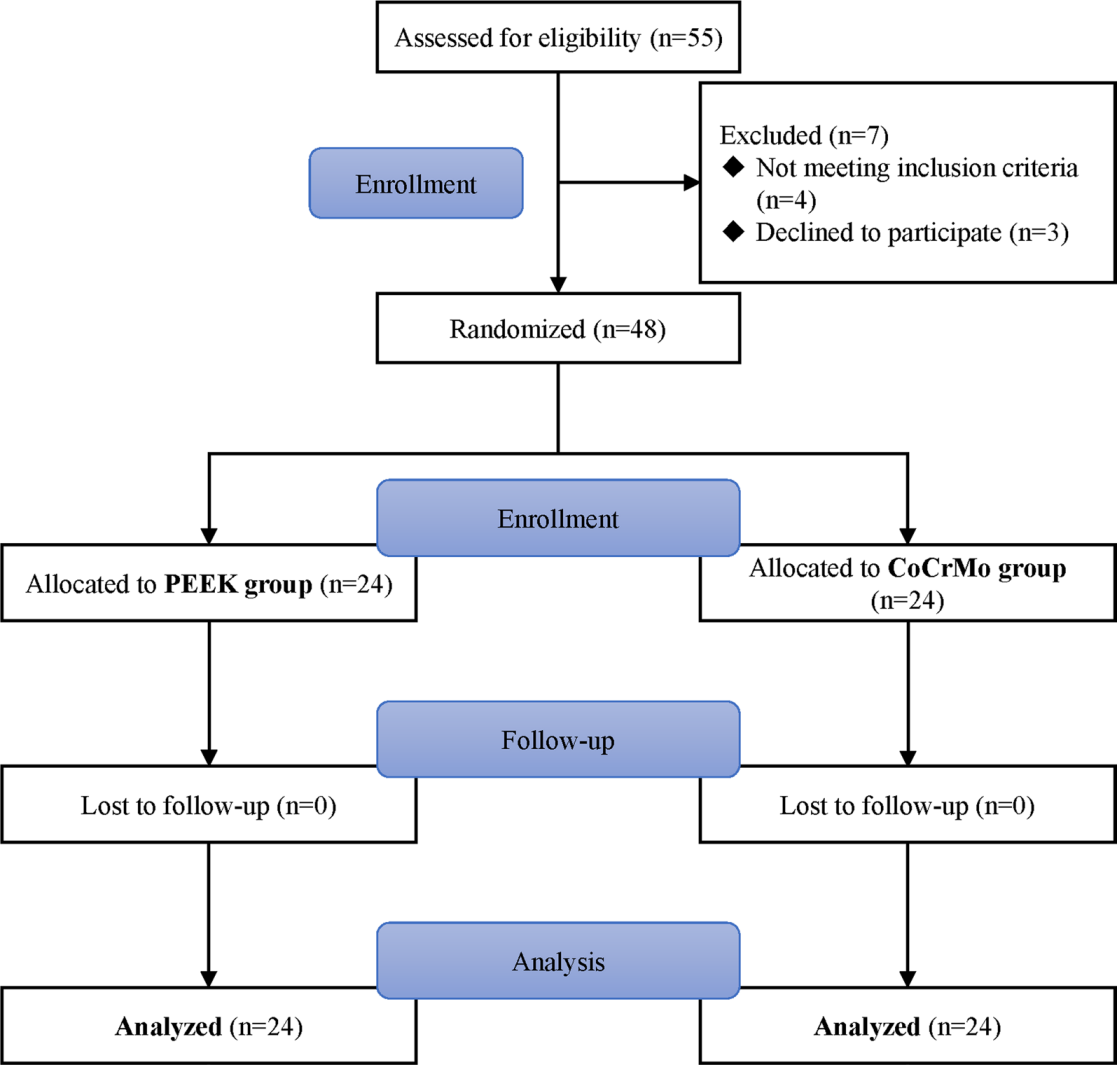
Flowchart of included participants

A total of 55 patients were enrolled in the trial, with 48 (37 males and 11 females) completing the study, as depicted in Fig. 3. Patients demonstrated comparable preoperative demographics, as illustrated in Table 1. No considerable differences were observed in terms of age, gender, BMI, ASA grade, or radiographic osteoarthritis grade. Additionally, surgical time, tourniquet use time, and length of hospital stay yielded analogous results between the two groups, with p-values of 0.37, 0.44, and 0.46, respectively.

**Table 1:** Comparison of baseline information and surgical information between the two groups

	PEEK group (*n* = 24)	CoCrMo group (*n* = 24)	*p value*
Gender (F/M)	17/7	20/4	0.30
Age (y)	66.75 ± 4.88	66.75 ± 5.78	1.00
BMI (kg/)	26.49 ± 3.68	25.54 ± 3.40	0.36
ASA grade (I/II/III, *n*)	0/21/3	0/20/4	0.68
Kellgren&Lawrence grade (III/IV, *n*)	6/18	8/16	0.53
Surgical time (min)	84.46 ± 14.26	80.92 ± 12.97	0.37
Tourniquet usage time (min)	64.25 ± 14.29	61.13 ± 13.31	0.44
Length of hospital stay (day)	5.63 ± 1.24	5.92 ± 1.47	0.46

**Fig. 4:**
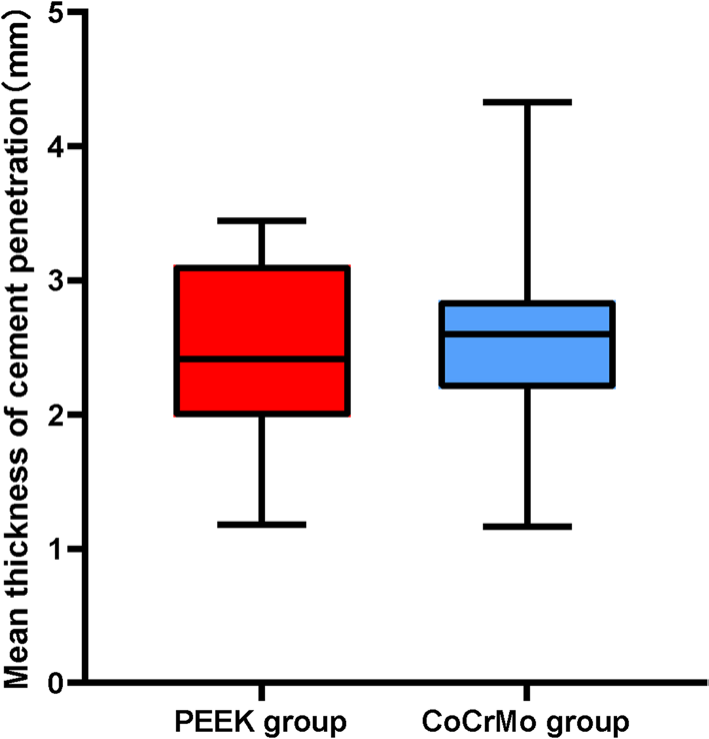
Box and whisker plot of mean cement mantle. Whiskers represent range, boxes represent 25th and 75th percentile, line represents median

There was no statistically significant difference in mean cement penetration on postoperative X-rays between the PEEK group (2.49 ± 0.61 mm) and the CoCrMo group (2.53 ± 0.68 mm) (*p* = 0.85) (Table 2, Fig. 4). Both the PEEK group and the CoCrMo group exhibited significant improvement in knee clinical outcomes and pain at the 1-year follow-up.

**Table 2:** Comparison of thickness of mean cement penetration between the two groups

	PEEK group (*n* = 24)	CoCrMo group (*n* = 24)	*p value*
Mean cement mantle penetration (mm)	2.49 ± 0.61	2.53 ± 0.68	0.85

However, knee clinical scores, functional scores, and visual analog scale (VAS) scores at 1-year postoperative did not show any statistically significant differences between the PEEK group and CoCrMo groups (*p* = 0.11; *p* = 0.21; *p* = 0.18, respectively) (Table 3).

**Table 3:** Comparison of knee clinical outcomes between the two groups

	PEEK group (*n* = 24)	CoCrMo group (*n* = 24)	*p value*
VAS score (preoperation)	7.33 ± 1.27	6.63 ± 1.66	0.10
VAS score (1 year postoperatively)	0.00 ± 0.00	0.13 ± 0.45	0.18
KSS knee score (preoperation)	59.00 ± 10.59	58.96 ± 9.60	0.99
KSS knee score (1-year postoperation)	89.21 ± 0.98	88.63 ± 1.44	0.11
KSS knee functional score (preoperation)	35.92 ± 16.05	28.54 ± 9.63	0.06
KSS knee functional score (1-year postoperation)	88.79 ± 7.27	85.96 ± 8.24	0.21

No patient was readmitted or underwent revision surgery. Out of all patients, three in the PEEK group and 6 in the CoCrMo group were diagnosed with deep-vein thrombosis, but no statistically significant difference was found between the groups (*p* = 0.46) (Table 4).

**Table 4:** Comparison of complications 1 year after surgery between the two groups

Complication	PEEK group (*n* = 24)	CoCrMo group (*n* = 24)	*p value*
DVT (*n*)	3	6	0.46
PE (*n*)	0	0	1.00
RLLs (*n*)	0	0	1.00
PJI (*n*)	0	0	1.00
PJF (*n*)	0	0	1.00
Delayed wound healing (*n*)	0	0	1.00

## Discussion

This study showed that the application of PEEK prostheses in primary TKA produced comparable cement penetration as the traditional CoCrMo prostheses, while simultaneously offering similar short-term clinical and functional outcomes without increased complications. This indicates that PEEK knee prostheses are a safe and effective prostheses that can provide excellent patient prognosis.

Between 2012 and 2021, the American Joint Replacement Registry (AJRR) collected data on 122,852 revision total knee arthroplasty (TKA) procedures, revealing that mechanical loosening of the prosthesis was the second-most common reason for knee revision surgery, accounting for 24.0% of cases [24]. For cemented implants, the implants–cement interface and the cement–bone interface are significant areas of contact. Previous studies have identified prosthetic debonding as a concern, as some tibial implants have ineffective strand locks at the prosthesis-bone cement interface, resulting in postoperative implant failure [25–27]. In an effort to address this issue, Jaeger et al. [28] conducted a cadaver study, implanting a tibial component with and without additional cement pockets in 15 fresh-frozen human leg pairs. The authors found that the additional cement pockets were biomechanically advantageous, improving the fixation performance of the implant. On the other hand, the stability of cemented implants is also determined by the cement–bone interface, which is influenced by factors such as the depth of penetration of the cement [18, 29]. The required depth of penetration for the cement to reach the first transverse trabeculae is between 2 and 3 mm [4]. Previous studies have reported varying levels of cement penetration, with averages ranging from 2.35 to 2.7 mm [18, 30]. The present findings were in line with previous studies, with an average depth of cement penetration between 2–3 mm. However, no statistically significant difference was observed between the PEEK and CoCrMo groups in terms of cement penetration.

As a copolymer compound, PEEK exhibits superior properties compared to metals, including reduced allergenicity, lighter weight, greater fatigue resistance, and chemical resistance [31, 32]. These unique characteristics make PEEK an attractive biomaterial for use in orthopedic applications, including the construction of femoral components, total hip replacements, and hip resurfacing [33]. In a recent study by Steinbergn et al. [34], the biomechanical properties of a tibia nail, a proximal humeral long plate, and a volar radial long plate were evaluated in vitro. The researchers reported similar mechanical properties between these PEEK devices and three commercially available titanium devices with similar designs.

Besides, several studies have investigated the performance of PEEK in different orthopedic implants using various testing methods and have reported positive findings regarding PEEK's mechanical properties, technical outcomes, and safety [35–38]. However, despite the favorable results, there is limited research on the use of PEEK in knee implants. The objective of this study was to evaluate the thickness of tibial cement penetration in patients with PEEK knee implants and compare them to those with conventional CoCrMo implants. The results showed that both the PEEK and CoCrMo knee implants resulted in cement penetration between 2 and 3 mm. There was no statistically significant difference in cement penetration between the two groups.

In this study, limited research had been reviewed on PEEK knee implants that has been conducted to date. Recent studies that investigated the feasibility and mechanical performance of PEEK-based knee implants had been summarized. Du et al. [39] conducted a preliminary study to investigate the feasibility and safety of a novel PEEK-based knee implant in a goat model. After 24 weeks of observation, the researchers found that the novel PEEK-polyethylene-bearing knee implant was feasible and safe in the goat model. The implant showed no signs of loosening, wear, or inflammation, indicating that PEEK may be a promising material for knee implants.

Ruiter et al. [40] used validated computational models to investigate the mechanical performance of PEEK femoral implants compared to CoCr implants. The researchers found that PEEK femoral implants could reduce periprosthetic stress shielding and increase strain energy density relative to preoperative bone and compared to CoCr. In a previous preliminary study conducted by Ruiter et al. [41], it was observed that, following 10 million cycles of walking gait, significant debonding was observed at the cement–implant interface for both PEEK and CoCr implants. Furthermore, no significant difference was found in the number of cement cracks between the two materials.

Additionally, Cowie et al. [42] investigated the potential use of injection-molded PEEK as a substitute for CoCr in femoral components for TKA, focusing on wear performance. The results showed that PEEK had comparable wear performance to CoCr, with a wear rate that remained linear over time. These preliminary findings suggest that PEEK may be a promising alternative femoral component material to CoCr in TKA. When introducing a new prosthetic material in humans, evaluating its clinical results and safety is of utmost importance, despite previous positive outcomes seen in animal studies. This study aimed to investigate the performance and effectiveness of PEEK knee joint implants, which can be used as an alternative to conventional CoCr alloy them in TKA. The results suggest that PEEK implant is a safe and effective choice that provides optimal patient outcomes, with minimal risk of complications. These data support the continued use and development of the PEEK biomaterial in the orthopedic field, with further investigations needed to confirm long-term results.

### Limitations

This study has several limitations. Firstly, this study presents a randomized controlled trial evaluating the cement penetration of the tibial component in TKA. However, the small sample size limits the analysis of the results. Secondly, only the cement penetration of the tibial component was analyzed, the femoral component cement penetration could not be assessed accurately on an anteroposterior radiograph, which is a limitation of the study. Further research is needed to investigate also the femoral component, with Roentgen Stereogrammetric Analysis potentially representing an effective tool to this aim. Thirdly, all surgeons were intraoperatively not blinded to the performed intervention. To reduce bias, the bone surface preparation, cement application, pressurization, and implantation of the components were standardized in both groups. Lastly, it should be noted that this study only evaluates cement penetration and cannot be extrapolated to TKA implant survivorship or longevity, further investigations evaluating the correlation between cement penetration and long-term outcomes of TKA implants are required.

## Conclusions

The PEEK knee prothesis can make a similar bone cement penetration satisfactory short-term clinical outcomes and safety compared to CoCrMo prothesis. Further studies are necessary to evaluate the potential benefits and long-term outcomes of PEEK TKA.

## Data Availability

The datasets used and/or analyzed during the current study are available from the corresponding author on reasonable request.

## Abbreviations

TKA: Total Knee Arthroplasty
PEEK: Poly-ether-ether-ketone
CoCrMo: Cobalt chromium molybdenum
DVT: Deep-vein thrombosis
TXA: Tranexamic acid
KSS: Knee Society Score
VAS: Visual analogue scale
PJI: Periprosthetic joint infection
PE: Pulmonary embolism
PJF: Periprosthetic joint fracture
RLLs: Radiolucent lines
